# Molecular Interactions between Methylene Blue and Sodium Alginate Studied by Molecular Orbital Calculations

**DOI:** 10.3390/molecules26227029

**Published:** 2021-11-21

**Authors:** Pasika Temeepresertkij, Michio Iwaoka, Satoru Iwamori

**Affiliations:** 1 Graduate School of Science and Technology, Tokai University, Hiratsuka 259-1292, Kanagawa, Japan; is107522@tsc.u-tokai.ac.jp; 2 Department of Chemistry, Tokai University, Hiratsuka 259-1292, Kanagawa, Japan; miwaoka@tokai.ac.jp

**Keywords:** methylene blue, sodium alginate, interaction position, molecular orbital calculations, binding affinity energy

## Abstract

A methylene blue (MB) indicator embedded in sodium alginate (SA) film was previously examined for detecting active oxygen species. In a previous study, spectrometry was used to identify and characterize the MB/SA complex. However, the decolorization mechanism was not fully assessed. In this study, our aim is to conduct computational calculations at the B3LYP/6-31G(d) level to clarify the exact types and positions of the interaction that cause the decolorization in MB. The results demonstrate that MB/SA interacts with carboxylates (-COO(superscript)-(superscript)) of SA and the N, C, and S atoms of MB, confirming previous experimental observations.

## 1. Introduction

Interactions between sodium alginate (SA) and methylene blue (MB) have been investigated for a long time. In particular, the chemical stability of the colorimetric indicator based on SA thin films and MB dyes upon exposure to active oxygen species (AOSs) has been examined [1]. Indeed, the MB/SA film indicators were discovered to be effective at detecting hydroxyl radicals (OH*) in the atmosphere. To detect OH* with higher oxidative abilities, it is necessary to stabilize MB by mixing it with a polymer matrix such that AOSs other than OH* cannot directly react with MB. SA, which has a high affinity for MB, was selected for this purpose because it stabilizes MB in its filmy matrix, thus preventing the attack of AOSs to MB. SA, a water-soluble polysaccharide biopolymer extracted from brown seaweed, has attracted the attention of researchers in recent decades [2,3]. In aqueous solutions, SA, a linear binary copolymer composed of β-D-mannuronic acid (M) and α-L-guluronic acid (G), exhibits an excellent film-forming ability and good moisture absorption, permeability, and high viscosity. SA has been extensively used in biomedical applications and the fabrication of new materials [4,5,6]. These biocomposite films are not only safe and environmentally friendly but also biocompatible, compensating for common inorganic materials employed for practical applications.

In a previous result [1], an AOS indicator obtained from uniform thin films was developed using MB-dyed SA, which can be generated under atmospheric conditions. Previous studies selectively detect OH* among various AOSs and develop AOS technology for sterilization and surface modification of polymer substrates. When the film was exposed to OH* with high humidity, decolorization did occur; however, the other AOSs did not decolorize the film under identical conditions. Experiments using a microplate reader were conducted to clarify the molecular interaction between MB and SA. The results demonstrated that both MB and SA share an ionic bond, as evidenced by a shift of UV absorbance corresponding to MB’s benzene ring in complexation with SA and that SA strongly stabilizes MB—enough to prevent it from interacting with atmospheric AOSs other than highly reactive OH*. Moreover, previous studies have revealed that SA and MB interact between SA’s carboxylates (‐COO^−^) and MB’s nitrogen or sulfur atoms [1]. However, the details of the intermolecular interactions were unclear.

The reaction of excited singlet oxygen, hydroxyl radicals (OH*), and ozone (O_3_) results in the decolorization of MB. However, the AOS indicators comprising a SA or pullulan film mixed with MB can be decolorized only by exposure to OH*, which has the strongest oxidizing ability among AOSs, but not by the exposure to excited singlet oxygen or O_3_. In this study, we conducted density functional theory (DFT) calculations to provide theoretical evidence for understanding how O_3_ causes MB decolorization and why the indicators made of SA or pullulan mixed with MB are not decolorized by the O_3_ exposure.

## 2. Calculation Methods

### 2.1. Materials

#### 2.1.1. Methylene Blue

**Figure 1:**
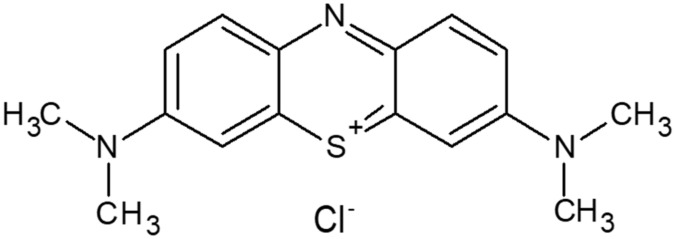
Structure of methylene blue (MB).

MB is a synthetic cationic thiazine dye with an amorphous nature in the solid state. The molecular formula is C_16_H_18_ClN_3_S as shown in Figure 1. It is called basic blue 9 [7,8] because it dissolves in water to form a dark blue-green solution that contains an MB cation and a chloride anion. This neutral ionic molecule was used for DFT calculations [9,10].

#### 2.1.2. Sodium Alginate

**Figure 2:**
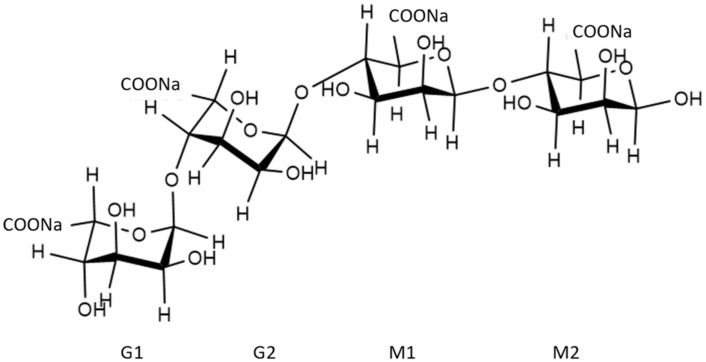
Structure of sodium alginate.

SA is composed of β-D-mannuronic acid (M) and α-L-guluronic acid (G) linked by 1–4 glycosidic bonds having the molecular formula (C_6_H_7_NaO_6_)_n_ [11]. In the calculation, this copolymer was simplified as a tetramer model, as shown in Figure 2.

### 2.2. Methods

#### Computational Details

For all calculations performed in this study, the Gaussian 09 program suite with a GaussView 5.0.9 interface was used [12,13]. All structures, including MS, SA, and their 1:1 complex, were completely optimized at the B3LYP/6-31G(d) level. The frequency calculation, which demonstrated no imaginary vibration frequency, confirmed the stationary point nature of the obtained structures.

For the MB/SA complex, we created 20 initial structures by randomly locating SA around the MB. These initial complex structures were completely optimized first at the B3LYP/6-31G(d) level. After the optimization, 10 stable structures were selected. For the 10 complex structures that were obtained, the intermolecular atomic distances between MB and SA were measured to identify the interaction positions. Single-point energy calculations were performed to calculate the binding energies for the MB/SA complex. The basis set superposition errors (BSSEs) were estimated using the Boys and Bernardi counterpoise method [14].

## 3. Results

### 3.1. Binding Energy

**Figure 3:**
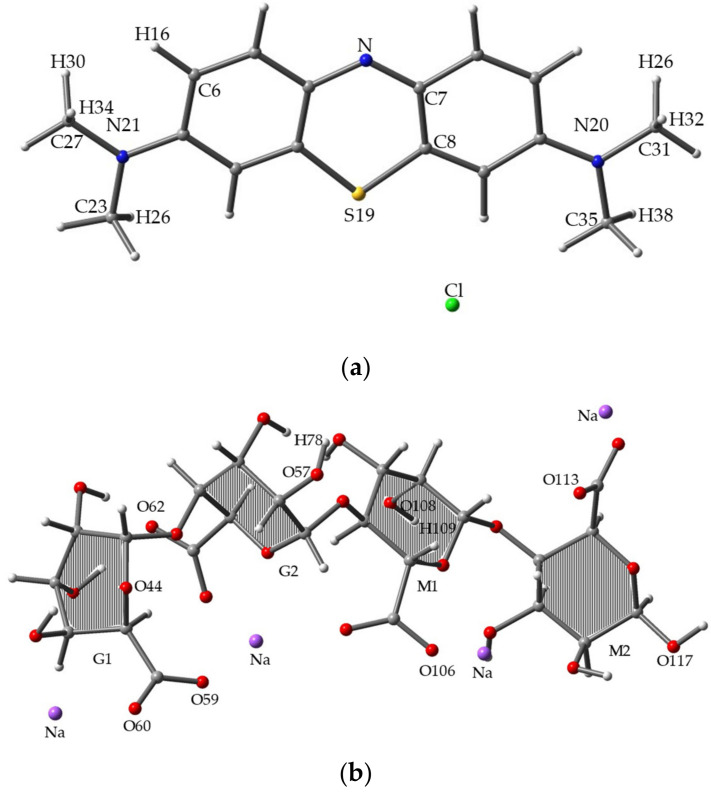

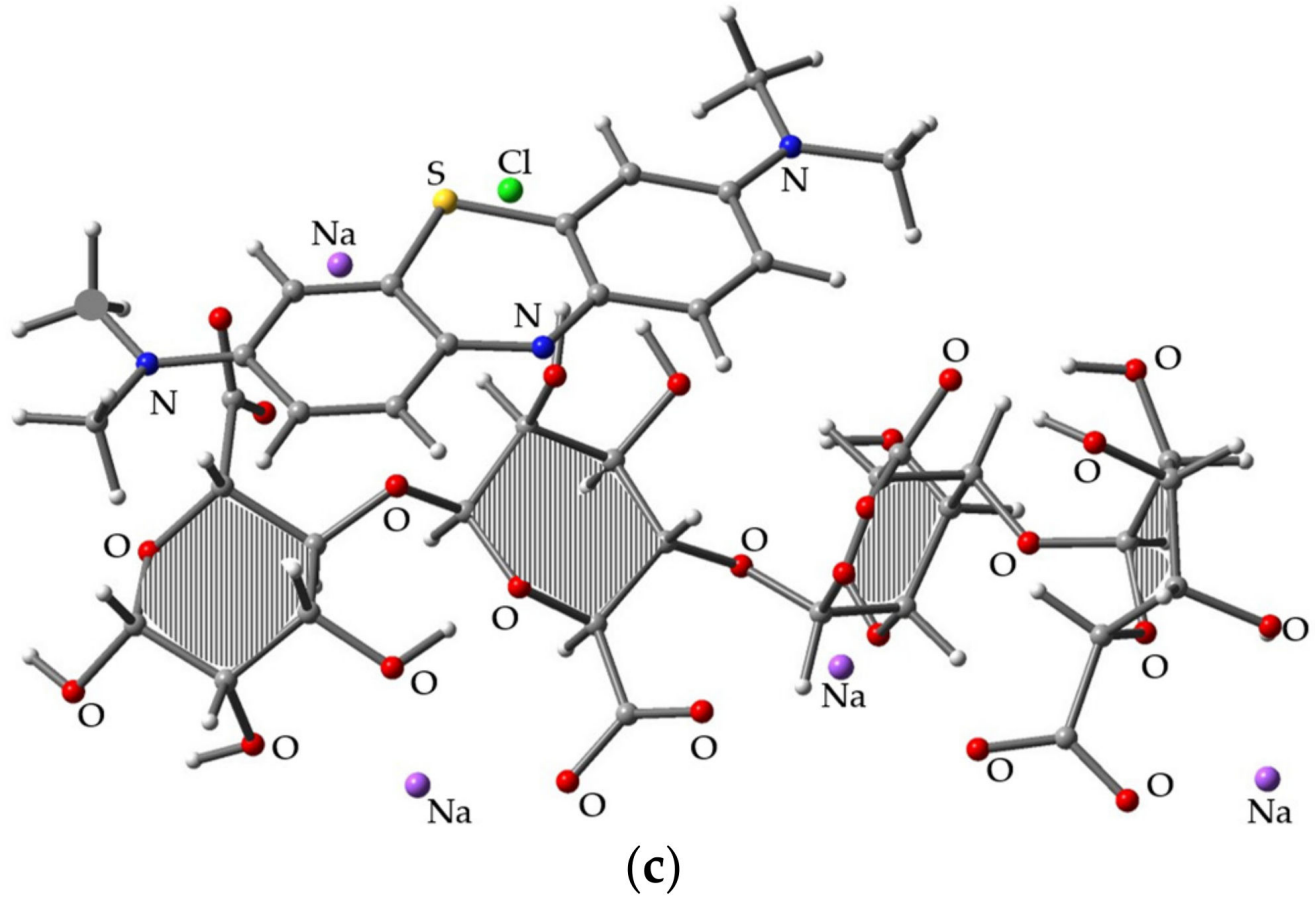
Optimized molecular structures for (**a**) MB, (**b**) SA, and (**c**) MB/SA at the B3LYP/6-31G(d) level of theory.

We calculated the binding energy at the B3LYP/6-31G (d) level of theory. The BSSE correction value was integrated with binding energy. Table 1 shows that MB/SA (1) has the largest binding energy (67.56 kcal/mol), making it the most stable complex structure. Figure 3c shows the results. However, MB/SA (10) has the smallest binding energy (38.13 kcal/mol). In terms of relative energy, it can be observed that the difference between MB/SA (1) and MB/SA (10) is 29.43 kcal/mol.

Figure 3a–c shows the 3D diagrams of molecular structures for MB, SA, and MB/SA (1). Figure 3a shows MB optimized at the B3LYP/6-31G(d) level, where the N, C, S, and H positions were analyzed. Figure 3b shows one of the stable structures of SA optimized at the B3LYP/6-31G(d) level, where the O and H positions were analyzed. Figure 3c shows the most stable structure obtained for MB/SA.

### 3.2. Atomic Distances

In the calculation results, we obtained 10 stable MB/SA complex structures and proceeded to determine the details of atomic interactions between MB and SA. As shown in Table 2, the interactions of the optimized molecular structures of MB/SA complex primarily occur between N, C, and S from MB and O from SA. Each data represent a short atomic distance corresponding to an ionic bond. The shortest distance of N---O is reported for MB/SA (3) between N22 and O117 (N---O = 2.96 Å). For MB/SA (10), the shortest distance of CH---O is 2.96 Å between C8 and O60. For MB/SA (6), the shortest distance of S---O is 3.23 Å between S19 and O60.

### 3.3. Simulation of Decolorization Mechanism of MB/SA with O_3_

**Figure 4:**
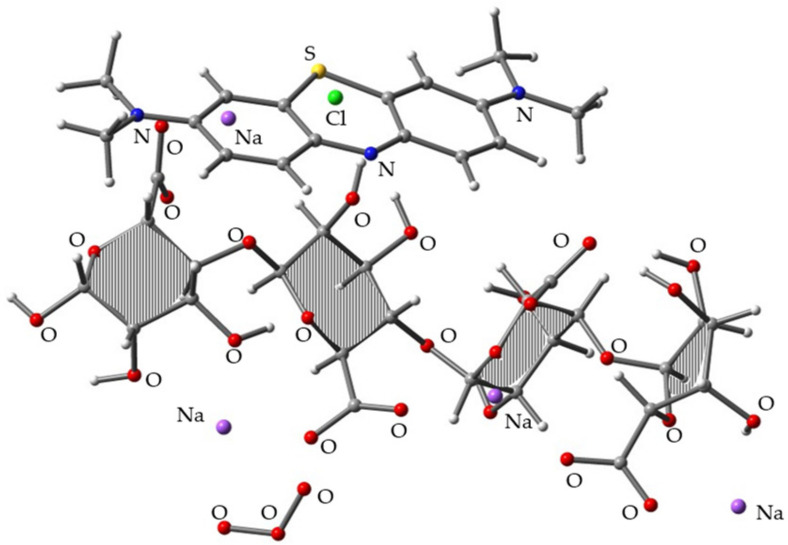
Optimized molecular structure obtained for the trimolecular complex of MB/SA (1) and O_3_ at the B3LYP/6-31G(d) level.

To build the trimolecular complexes among MB, SA, and O_3_, we used the most stable MB/SA complex structure (i.e., MB/SA (1)) and aimed to position O_3_ as close to SA as possible since we considered that the SA acts as a protection layer for MB. After full geometry optimization at the B3LYP/6-31G(d) level, ~10 distinct complex structures were obtained. Figure 4 shows the most stable complex. The binding energy of the three components was 78.97 kcal/mol, which is 11.41 kcal/mol higher than the MB/SA (1) binding energy.

Table 3 shows the comparison of the HOMO and LUMO energy levels of O_3_, MB, SA, pullulan, MB/SA, and MB–pullulan obtained at the same calculation level. The results can be used to analyze the thin film’s decolorization mechanism [1]. As shown in Table 3, the LUMO level of O_3_ is low (−4.90 eV). If a substrate’s HOMO level is above this level, oxidation will easily occur. MB’s HOMO is −4.08 eV, indicating that it can be oxidized with O_3_. However, the HOMO levels of SA and pullulan are lower than the LUMO level of O_3_, indicating that the oxidation of SA and pullulan with O_3_ is difficult. Nevertheless, when SA and pullulan are combined with MB, creating MB/SA and MB-pullulan complexes, we observed that the HOMO level of MB decreases to −5.73 and −5.57, respectively, strongly suggesting the prevention of MB from O_3_ oxidation in the matrix of SA and pullulan.

## 4. Discussion

This study discussed the molecular interactions between MB and SA to understand the decolorization behavior of MB/SA complexes with AOSs. Therefore, the results are reliable and can be effectively used to develop new indicators for AOS detection. An advantage of simulation is that it enables us to examine the complex’s 3D molecular structure in a thin film. This 3D molecular structure leads us to a more accurate interaction model because it can be performed freely multiple times, minimizes human error, and shows precise numerical results. The results demonstrate that the MB/SA structure was analyzed for the molecular interactions, which are important for the AOS exposure. The simulation program shows the possibility of intermolecular interactions and indicates that both types of polymers are effective in uniform thin films for AOS detection. HOMO and LUMO show that pullulan is more effective in protecting MB than SA, which corresponds to the previous study.

In this study, we investigated the intermolecular interactions between MB and SA as well as the decolorization behavior of the MB/SA complex with AOSs. We performed density functional theory (DFT) calculations for the bimolecular and trimolecular complexes among MB, SA, and O_3_. Thus, the molecular reasons for the MB/SA film’s resistance to decolorization during O_3_ exposure were discovered. We reported that the HOMO of MB is higher than the LUMO of O_3_, indicating the fast oxidation of MB with O_3_, whereas the HOMO of MB mixed with SA was lower than the LUMO of O_3_. We reported a similar behavior of the MB complex with pullulan. In our previous study [1], we presented the development of a color indicator, which is composed of an SA or pullulan thin film embedding an MB reporter, for detecting AOSs with enhanced oxidative ability. We assumed that the SA or pullulan protects MB based on the fact that AOSs with a low oxidative ability cannot decolorize the MB. In this study, we focused on determining the most stable structure of the MB/SA complex to explain our earlier observations.

In a previous study, we examined the chemical reactions and decolorization mechanism associated with the exposure of the optimized MB–pullulan structures to OH* [15,16,17,18]. We show the results that provided insight into possible interaction modes that allow AOS detection under various conditions. Pullulan is a natural water-soluble polysaccharide with excellent film-forming properties but has a large and complex structure. Furthermore, we reported that pullulan has a helix structure and is considered a macromolecule. Here, the stabilization of MB is achieved by mixing the dye with pullulan, which is required for removing MB’s color because the macromolecules can effectively protect the MB molecules from AOSs. Therefore, it was a challenge to identify the interaction modes with MB.

In MB/SA complex structures, the most stable binding site for MB in the analysis is the N atom, C atom, and S atom. We reported that, at the molecular arrangement structure, the ‐COO^−^ carboxylates of the SA-optimized molecular structures were more likely to interact with OH for SA. The binding affinity of molecules is the total energy released by the thrust and gravitational force between the nucleus and electron, as well as the action force between two electrons. The total energy of molecules can be obtained using molecular structure calculations. If the result presents a stable structure, the binding energy will be negative. Furthermore, the same molecule with the lowest total energy is more stable than the molecule with the highest total energy. Binding energy here is defined as the difference between the total energy of MB-SA and that of the reactants (MB and SA). Using a finite basis set, a BSSE occurs when the atoms of interacting molecules approach one another.

## 5. Conclusions

In this study, we performed DFT calculations at the B3LYP/6-31G(d) level of theory for MB, SA, and MB/SA. The results demonstated that the stable MB/SA complex structures possess intermolecular interactions between the carboxylates (‐COO^−^) of SA and the N, C, and S atoms of MB. The result was consistent with previous studies [1]. The calculation for MB/O_3_ and MB/SA or pullulan/O_3_ complexes showed that O_3_ can decolorize MB; however, the indicators comprising SA or pullulan mixed with MB cannot be decolorized in O_3_ exposure. Thus, the suitability of selecting SA or pullulan as a polymer matrix to develop AOS detectors was validated.

## Figures and Tables

**Table 1:** Binding energy (in kcal/mol) for the MB/SA complexes characterized at the B3LYP/6-31G(d) level.

Structure Name	Binding Energy (kcal/mol)	Relative Energy (kcal/mol)	BSSE (kcal/mol)
MB/SA (1)	67.56	0	11.53
MB/SA (2)	67.50	0.06	11.55
MB/SA (3)	49.58	17.98	12.27
MB/SA (4)	49.09	18.47	7.06
MB/SA (5)	47.06	20.5	6.16
MB/SA (6)	44.36	23.2	10.05
MB/SA (7)	42.49	25.07	8.20
MB/SA (8)	42.49	25.07	8.20
MB/SA (9)	39.08	28.48	10.22
MB/SA (10)	38.13	29.43	9.31

**Table 2:** The atomic distances between N, C, and S from MB and O from SA for the complexes obtained at the B3LYP/6-31G(d) level.

Structure Name	Position	N---O (Å)	Position	C-H---O (Å)	Position	S---O (Å)
MB/SA (1)	N22,O108,H109	3.91	H32,C31,062	3.24	S19,O108	3.48
MB/SA (2)	N22,O60	3.51	H30,C27,O106	3.46	S19,O60	3.27
MB/SA (3)	N20,O117	2.96	H38,C35,059	3.26	S19,O113	3.71
MB/SA (4)	N20,O106	4.07	H26,C23,O106	3.45	S19,O113	4.88
MB/SA (5)	N20,O57,H78	3.18	H30,C27,O57	3.19	S19,O113	5.94
MB/SA (6)	N20,O57,H78	3.74	H16,C6,O106	3.53	S19,O60	3.23
MB/SA (7)	N21,O60	3.27	H38,C35,O60	3.13	S19,O44	4.62
MB/SA (8)	N21,O60	3.27	H38,C35,O60	3.13	S19,O44	4.62
MB/SA (9)	N22,O60	3.46	C7,O60	3.17	S19,O60	3.27
MB/SA (10)	N22,O60	3.51	C8,O60	2.96	S19,O60	3.27

**Table 3:** HOMO and LUMO energy levels at the B3LYP/6-31G(d) level.

Structure Name	HOMO (eV)	LUMO (eV)	HOMO-LUMO (eV)
O_3_	−8.98	−4.90	−4.08
MB	−4.08	−2.99	−1.09
SA	−5.40	−2.05	−3.35
Pullulan	−6.73	0.81	−7.53
MB/SA	−5.73	−3.32	−2.41
MB/pullulan	−5.57	−3.48	−2.09

## Data Availability

Not applicable.

## Sample Availability

Samples of the compounds methylene blue, sodium alginate, and pullulan are available from the authors.

## References

[1] Yenchit S., Yamanaka H., Temeeprasertkij P., Oda Y., Kanie O., Okamura Y., Inazu T., Iwamori S. (2020). Chemical stability of a colorimetric indicator based on sodium alginate thin film and methylene blue dye upon active oxygen species exposure. Jpn. J. Appl. Phys..

[2] Wang Z., Yang H., Zhu Z. (2019). Study on the Blends of Silk Fibroin and Sodium Alginate: Hydrogen Bond Formation, Structure and Properties. Polymer.

[3] Lan W., He L., Liu Y. (2018). Preparation and Properties of Sodium Carboxymethyl Cellulose/Sodium Alginate/Chitosan Composite Film. Coatings.

[4] Harnsilawat T., Pongsawatmanit R.J.D. (2006). McClements. Characterization of β-Lactoglobulin–Sodium Alginate Interactions in Aqueous Solutions: A Calorimetry, Light Scattering, Electrophoretic Mobility and Solubility Study. Food Hydrocoll..

[5] Yue Y., Wang X., Han J., Yu L., Chen J., Wu Q., Jiang J. (2018). Effects of nanocellulose on sodium alginate/polyacrylamide hydrogel: Mechanical properties and adsorption-desorption capacities. Carbohydr. Polym..

[6] Xiao C., Liu H., Lu Y., Zhang L. (2001). Blend Films from Sodium Alginate and Gelatin Solutions. J. Macromol. Sci. Part A.

[7] Warren C. (2008). Rapid Measurement of Chlorophylls with a Microplate Reader. J. Plant Nutr..

[8] Raposo F., De La Rubia M.A., Borja R. (2009). Methylene blue number as useful indicator to evaluate the adsorptive capacity of granular activated carbon in batch mode: Influence of adsorbate/adsorbent mass ratio and particle size. J. Hazard. Mater..

[9] Zhang G., Musgrave C.B. (2007). Comparison of DFT Methods for Molecular Orbital Eigenvalue Calculations. J. Phys. Chem. A.

[10] Nori-Shargh D., Amini M.M., Jamehbozorgi S. (2003). Ab Initio Study of Ring Flipping of the Overcrowded Peri-Substituted Naphthalenes. Phosphorus Sulfur Silicon Relat. Elem..

[11] Fu S., Thacker A., Sperger D.M., Boni R.L., Buckner I.S., Velankar S., Munson E.J., Block L.H. (2011). Relevance of Rheological Properties of Sodium Alginate in Solution to Calcium Alginate Gel Properties. AAPS PharmSciTech.

[12] Tabti C., Benhalima N. (2015). Molecular Structure, Vibrational Assignments and Non-Linear Optical Properties of 4,4’ Dimethylaminocyanobiphenyl (DMACB) by DFT and ≪i≫Ab Initio HF Calculations. Adv. Mater. Phys. Chem..

[13] Temeepresertkij P., Yenchit S., Iwaoka M., Iwamori S. (2020). Interactions between Methylene Blue and Pullulan According to Molecular Orbital Calculations. IEEJ Trans. Fundam. Mater..

[14] Boys S.F., Bernardi F. (1970). The calculation of small molecular interactions by the differences of separate total energies. Some procedures with reduced errors. Mol. Phys..

[15] Iwamori S., Nishiyama N., Oya K. (2016). A colorimetric indicator for detection of hydroxyl radicals in atmosphere using a methylene blue dye based on nafion film. Polym. Degrad. Stab..

[16] Hosoya K., Yenchit S., Tadokoro Y., Oya K., Iwamori S. (2018). Improved Singlet Oxygen Detection Sensitivity in Electron Spin Resonance Using a Spin-trap Agent Incorporated into a Water-soluble Polymer Film. Chem. Lett..

[17] Oya K., Hosoya K., Suto T., Iwamori S. (2019). Surface modification of polydimethylsiloxane by exposure of active oxygen and ultraviolet lights to improve cell adhesion. Trans. JSME.

[18] Yenchit S., Tadokoro Y., Iwamori S. (2019). Measuring Active Oxygen Species Across a Nonwoven Fabric Using a Pullulan-mixed Methylene Blue Thin Film and Electron Spin Resonance. IEEJ Trans. Sens. Micromach..

